# Emerging implications of *N*6-methyladenosine in prostate cancer progression and treatment

**DOI:** 10.1038/s41420-025-02680-w

**Published:** 2025-08-19

**Authors:** Junyan Xu, Dajun Gao, Changjie Ren, Zhong Wang, Fuwen Yuan, Yanting Shen

**Affiliations:** 1 School of Gongli Hospital Medical Technology, University of Shanghai for Science and Technology, Shanghai, China; 2 Department of Urology, Shanghai Ninth People’s Hospital Affiliated Shanghai Jiaotong University School of Medicine, Shanghai, China; 3 Department of Urology and Andrology, Gongli Hospital of Shanghai Pudong New Area, Shanghai, China; 4 Laboratory for Research in Urology and Andrology, Gongli Hospital of Shanghai Pudong New Area, Shanghai, China; 5 The Center for Cancer Research, School of Integrative Medicine, Shanghai University of Traditional Chinese Medicine, Shanghai, China

**Keywords:** Targeted therapies, Prostate cancer

## Abstract

RNA modifications are widely distributed in almost all types of RNA, including mRNA, rRNA, miRNA, circRNA, and lncRNA, which are deeply involved in disease initiation and progression and are emerging therapeutic targets in diseases such as cancer, among which *N*6-methyladenosine (m6A) is the most abundant mRNA modification. Accumulating studies have demonstrated the critical role of m6A during cancer progression and its therapeutic potential in prostate cancer, which is one of the most common malignancies in men worldwide. Here, we reviewed the emerging roles of m6A regulators, including readers, writers, and erasers, and the downstream m6A-modified mRNA and noncoding RNA in prostate cancer. We also discussed the therapeutic potential of targeting m6A in prostate cancer and summarized the emerging agents and technologies, such as the cutting-edge CRISPR-Cas13 in prostate cancer treatment by targeting m6A regulatory pathways. At last, we elucidated the perspective of developing efficient and specific RNA targeting agents and technological platforms to provide new strategies for treating prostate cancer by targeting RNA modifications.

## Introduction

Prostate cancer (PCa) is one of the most commonly diagnosed cancers in men worldwide [1]. Surgical removal of the prostate, radiation, chemotherapy, or hormone therapy are the main treatments for the vast majority of patients. Despite the improvement of these therapies, approximately 10% of patients are diagnosed with metastatic prostate cancer, which carries a 5-year survival rate of only 37% [2]. The prognosis of early-stage PCa is good with surgery and androgen deprivation therapy (ADT), while most patients eventually develop metastatic castration-resistant prostate cancer after ADT [3]. Metastasis is one of the most important factors responsible for death, and the survival rate decreases significantly if the tumor has metastasized since diagnosis [4]. In addition to surgical treatment and ADT, most chemotherapy drugs have narrow therapeutic indexes, poor pharmacokinetics, and non-selective distribution in vivo, leading to high drug toxicity and side effects. Therefore, it is urgent to better understand the molecular mechanism that regulates the metastasis of PCa and find new targets for PCa cells with reduced side effects.

Post-transcriptional modifications are widely distributed across mRNA, rRNA, miRNA, and lncRNA, which are deeply involved in the occurrence and development of tumors and are emerging therapeutic targets, among which *N*6-methyladenosine (m6A) is the most abundant mRNA modification [5, 6]. In molecular mechanisms, m6A is involved in almost all steps of RNA metabolism, such as RNA degradation, splicing, export, folding, mRNA translation, etc.[7]. m6A has emerged as a multifaceted controller for gene expression regulation, mediated through its regulatory proteins, including writers, readers, and erasers [8]. It plays an important role in gene expression regulation [9]8, animal development [10], and human diseases [11]. The regulators in m6A pathways include “writers” and “erasers” that respectively install and remove the methylation, and “readers” that recognize methylation status [8, 12]. The methyltransferase complex contains two catalytic components, METTL3 (Methyltransferase-like 3, also known as MTA70, METTL14) and the accessory WTAP (Wilm’s tumor 1-associated protein) [13]. As demethylases, ALKBH5 and FTO can reverse m6A methylation, assisted with m6A-binding proteins, including members of the YTH domain family and the h*eterogeneous nuclear* ribonucleoprotein (HNRNP) family [1, 14]. During prostate progression, some writer proteins can inhibit the degradation of relevant mRNA by targeting m6A, thus affecting cancer progression [15]. Moreover, m6A-modified ncRNAs, such as lncRNA, circRNA, miRNA, and siRNA, in PCa can participate in the formation process of RNA complexes, thereby affecting the progression of prostate cancer [14].

Given the critical implications of m6A in cancer progression, emerging efforts are being made to develop agents and technologies for targeting the oncogenic m6A regulators and events. Notably, small molecules, especially agents derived from traditional medicines, are being used as m6A-targeted anticancer drugs, which treat the disease by targeting prostate cancer-related m6A. In addition, emerging technologies such as CRISPR-Cas9 and CRISPR-13 are also being applied with anti-tumor diagnostics and therapies, which have achieved great advantages and effectiveness, especially by specifically targeting RNA modifications such as m6A. Studies have shown that CRISPR-Cas13 can target m6A for the treatment of m6A dysregulation-related diseases, and it is reasonable to expect that this technology can be better integrated with m6A-targeted treatment of prostate cancer in the future.

In this review, we summarized the roles and mechanisms of m6A regulators and m6A-modified oncogenic or tumor-suppressor RNAs in PCa and discussed and prospected the therapeutic potential of the agents and CRISPR-Cas13 platforms for the treatment of prostate cancer by targeting m6A.

## M6A regulators in prostate cancer

**Fig. 1:**
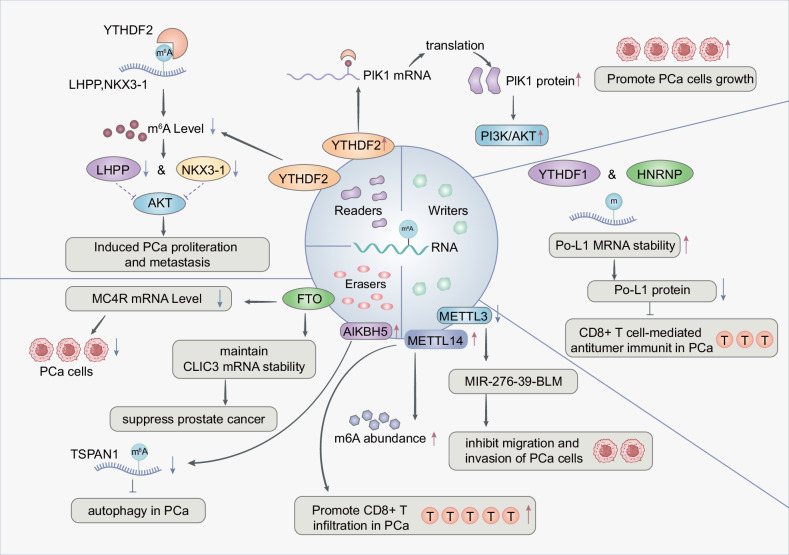
Molecular mechanisms of m6A writers, erasers, and readers in prostate cancer. The m6A Reader protein YTHDF2 promotes prostate cell proliferation by modulating the phosphorylation of downstream AKT signaling. YTHDF1 enhances anti-tumor immunity in prostate cancer by regulating mRNA stability and thereby affecting protein expression in T cells. The Eraser proteins FTO and ALKBH5 modulate the m6A methylation level of TSPAN1, leading to the suppression of autophagy in prostate cancer cells. The Writer protein METTL14 increases m6A abundance, thereby promoting T cell infiltration into the tumor microenvironment. In contrast, downregulation of METTL3 has been shown to inhibit prostate cancer progression.

M6A is a dynamic and reversible RNA modification and is one of the most frequently reported RNA modifications in cancer. The m6A is added, removed, or recognized by the corresponding writer, eraser, and reader, respectively. Dysregulated expression or activity of “writers” and “erasers” determines the abnormal level of m6A in cancer, and “readers” are mainly responsible for the recognition of the targeted RNA m6A modification. Briefly, the “writers” mainly include METTL3, METTL14, and cofactor WTAP [9, 16–18]. The YTH domain-containing proteins including YTHDF1, YTHDF2, and YTHDF3 are the “readers”[8]. In addition, ALKBH5 and FTO are the main “erasers”, which is now clear that they demethylate m6A. The basic process of m6A modification is that it is installed by m6A methyltransferase, removed by m6A demethylase, and recognized by m6A reading molecules, thereby regulating RNA metabolism. Many studies have found that m6A is widely involved in PCa initiation, progression, and resistance through various molecular mechanisms [16, 19] (Fig. 1).

### m6A “writers” in prostate cancer

The m6A “writers” function to promote the methylation of targeted RNAs. There are seven “writers” reported, including METTL3, METTL5, METTL14, WTAP, RBM15, ZC3H13, and VIRMA [17]. Diverse writers have disparate effects on different types of cancer by regulating the status of target RNA m6A [18]. Dysregulation of METTL3 can promote or inhibit cancer progression through distinctive mechanisms in different cancers. It has been demonstrated in pancreatic cancer that cigarette smoke condensation induces hypomethylation of the METTL3 promoter, followed by recruitment of the transcription factor NFIC to induce METTL3 overexpression and promote cancer progression [20]. The gut microbial metabolite butyrate is reported to downregulate METTL3 expression and inhibit the development of colorectal cancer [21]. Cui et al. reported that METTL3 knockdown in liver cancer significantly inhibits HB cell proliferation, migration, and invasion by regulating Wnt/β-catenin pathway-related proteins [22]. Mechanistic studies revealed that METTL3 may exert an oncogenic role by targeting and regulating the m6A modification of oncogenes such as Myc and cyclin D1 in PCa [23]. Specifically, the oncogenic function of METTL3 depends on its methyltransferase catalytic activity, as a mutant METTL3 without catalytic activity cannot methylate the Myc mRNA and lose its tumorigenesis-promoting role [23]. Cai et al. found that the reduction of METTL3 inhibited the SHH-GLI1 signaling axis, decreased mRNA levels of the downstream targets, such as c-Myc and cyclin D1, and significantly decreased PCa cell survival and colony formation capacity [24]. By lowering the amounts of ubiquitin-specific protease 4 (USP4) protein, METTL3 causes the ELAVL1 (embryonic lethal, abnormal vision, Drosophila-like 1) protein to degrade and increases the expression of Rho GDP dissociation inhibitor (GDI) alpha protein (ARHGDIA), thus promoting PCa cells migration and invasion [25, 26]. The highly expressed METTL3 reduces the ELAVL1 protein level by inducing the degradation of ELAVL1 protein in PCa cells and increases the expression of downstream invasion and migration-related protein ARHGDIA, and significantly upregulates the mRNA level, thus promoting the proliferation and migration of prostate cancer [26]. METTL3 can upregulate plasmacytoma variant translocation 1 (PVT1) and regulate the miR-27b-3p/bloom syndrome protein (BLM) axis to promote the progression of PCa [27]. Li et al. found that castration of m6A and METTL3 activated the ERK pathway and promoted ADT resistance in prostate cancer [28]. These studies collectively indicated the oncogenic role of METTL3 in cancers.

Another m6A writer, METTL14, is both a tumor suppressor and an oncogene in different conditions. The dysregulation of METTL14 is related to tumorigenesis, proliferation, metastasis, and invasion [29]. Knockdown of METTL14 in macrophages promotes tumor development in colorectal cancer [30], and knockdown of miR-126, the downstream target gene of METTL14, can also promote tumor metastasis in metastatic liver cancer [29]. In contrast, upregulation of METTL14 was correlated with poor prognosis in PCa patients, and knockdown of METTL14 inhibited tumor proliferation both in vitro and in vivo by regulating the expression of Thrombospondin 1 (THBS1) in an m6A-dependent manner, resulting in the recruitment of YTHDF2 to recognize and degrade THBS1 mRNA [31]. Besides, recent studies demonstrated that WTAP, a regulatory subunit of methyltransferase, is an oncogene that closely influences tumor progression, whose absence reduces the RNA-binding capacity of methyltransferase [32]. In general, WTAP is upregulated in a variety of cancers, including liver cancer, esophageal cancer, AML, and osteosarcoma [33–39]. circPDE5A interacts with WTAP to inhibit prostate cancer metastasis and block certain activities of m6A [40]. Other “writers” are also reported to correlate with PCa tumorigenesis or progression. For example, ZC3H13 demonstrated a marked decrease in PCa, while RBM15B and RBM15 showed high expression in PCa tissues [41].

### m6A “readers” in prostate cancer

Numerous m6A “readers” have been reported so far, including YTHDF1, YTHDF2, YTHDF3, YTHDC1, YTHDC2, RBMX, LRPPRC, IGF2BP3, IGF2BP2, IGF2BP1, HNRNPC, HNRNPA2B1, FMR1, and ELAVL1 [15]. As an important m6A reader, YTHDF2 was previously demonstrated to induce targeted mRNA decay [42, 43]. YTHDF2 mediated the mRNA degradation of LHPP and NKX3-1 by reading m6A-modified sites in m6A-dependent manners [44]. Recent research has established that m6A readers impact the incidence and progression of PCa. Upregulated YTHDF2 was involved in PCa proliferation and migration by regulating m6A levels and phosphorylated AKT signal pathways [44]. Another m6A reader, YTHDF1, also acts as an oncogene in PCa by regulating several downstream m6A-modified targets. YTHDF1 boosts the expression of m6A-modified polo-like kinase 1 (PLK1) by the PI3K/AKT signaling pathway to drive the progression of PCa [45]. YTHDF1 can also promote prostate cancer progression by regulating TRIM44 m6A modification and degradation [46]. At the same time, YTHDF1 can also repress the CD8+ T cell-mediated anti-cancer immunity and ferroptosis in prostate cancer in an m6A/PD-L1 manner [47]. SLC12A5 interacts with YTHDC1 and upregulates the transcription factor HOXB13, thereby promoting prostate cancer progression [48].

In addition to the oncogenic roles of YTHDF1 and YTHDF2, knocking down HnRNPL can reduce the stability of YY1 mRNA in CRPC and inhibit the migration of tumor cells [49]. It can also induce autophagy through the cirCCSPP1-mir-520h-EGR1 axis to promote the development of prostate tumors [50]. HnRNPH/F ablation leads to G2/M cell cycle arrest, induces apoptosis of prostate cancer cell lines, and inhibits the proliferation and migration of cancer cells [50, 51]. Other readers such as ELAVL1, HNRNPA2B1, HNRNPC, RBMX, YTHDC2, YTHDF1, and YTHDF2 were also expressed at a high level in PCa tissues, but FMR1, IGF2BP2 were expressed at low levels in PCa tissues [52]. Studies have found that ELAVL1 interacts with YTHDC1 and IGF2BP1, which play a synergistic regulatory role in tumors [53, 54]. However, the functions of these readers in PCa need to be further explored.

### m6A erasers in prostate cancer

Demethylases, also known as “erasers”, include FTO and ALKBH5, which function to remove the m6A methyl group from RNA and are also reported to be implicated in prostate cancer progression. FTO, as a well-studied m6A demethylase, is associated with a greater risk of endometrial cancer, breast cancer, thyroid cancer, and pancreatic cancer [55–58]. The sequences of FTO were analyzed, and the results showed the distant homology of FTO and ALKB dioxygenase families [59]. FTO is a nucleic acid demethylase that can act on both DNA and RNA [60]. The expression of FTO in tumor cells is lower than that in normal cells. Downregulation of FTO can promote the progression of PCa by targeting MCR4 in the way of m6A modification, while overexpression of MCR4 can promote the malignant progression of prostate cancer cells [61]. FTO knockout increases m6A methylation of p53 transcriptional targets, including FAS, TP53INP1, and SESN2, and induces cycle arrest and apoptosis in PCa [62]. Recent studies also suggested that FTO suppresses PCa growth and migration by lowering the decay of CLIC4 mRNA and that CLIC4 is a functional target of FTO-mediated m6A modification [63].

Furthermore, ALKBH5 is an endogenous m6A demethylase, whose knockdown or overexpression led to an increase or decrease of several critical oncogenic RNA m6A in cancers. ALKBH and most AlkB homologs have α-Kg conservative asparagine residue with hydrogen bonds, but ALKBH7 has no such structure [64]. Unlike FTO, ALKBH is not active against m6A [65]. ALKBH5 was demonstrated to be upregulated in some cancers, such as glioblastoma stem cells [66, 67], breast cancer stem cells [68], colorectal cancer, esophageal cancer, thyroid cancer, gastric cancer, acute myeloid leukemia, and prostate adenocarcinomas [68–70]. Some studies have found that most of the targets of FTO may not be mRNAs, but snRNA [71]. Future research should focus on identifying the exact roles and mechanisms of these downstream effectors in cancers.

## Dual roles of mRNA m6A modifications in cancers

Increasing evidence shows that m6A of different downstream RNAs plays distinct roles in cancer [72, 73]. On the one hand, m6A modification enhances oncogenes’ stability or translation efficiency or downregulates the tumor suppressors’ mRNA activity to promote cancer initiation and progression [74, 75]. On the other hand, m6A events may result in the corresponding oncogenic mRNA degradation, which thereby inhibits cancer proliferation, migration, and invasion [24, 44, 76–79].

### Tumor promotion roles of mRNA m6A modifications

In breast cancer, upregulation of m6A modifications in MYB, BCL2, and PTEN can enhance the protein-binding ability and translation efficiency of RNA, leading to tumorigenesis [80]. Similarly, in hepatocellular carcinoma, excessive m6A modification of the tumor suppressor gene SOCS2 reduces mRNA stability and accelerates its degradation, leading to tumor progression [81]. In addition, upregulated m6A modification on HBXIP and MAGT3 in breast cancer also promotes cancer development [82]. In PCa, PCAT6 directly interacts with IGF2BP2 and upregulates the stability of IGF1R mRNA. Overexpression of PCAT6 or IGFBP2 increased the stability of IGF1R mRNA, and IGF1R overexpression in turn enabled the inhibitory role of PCAT6 on the proliferation of PCa [74]. Some studies indicated that METTL3 influences the activity of the Wnt/β-catenin pathway through m6A methylation of lymphoid enhancer-binding factor 1 (LEF1) mRNA, thereby promoting the progression of PCa [75]. In summary, RNA hyper-m6A of several mRNAs accelerates tumor progression in PCa.

### m6A functions as a tumor suppressor

On the other hand, mRNA m6A may also suppress tumor growth in PCa through several targets. NKX3-1 has been reported as a tumor suppressor whose functions are closely linked to its mRNA m6A modification status, and loss of NKX3-1 contributed to prostate carcinogenesis and tumor progression [76–78]. Knocking down both YTHDF2 and METTL3 induced NKX3-1 expression and inhibited NKX3-1 m6A-dependent AKT phosphorylation in PCa [44]. Besides, m6A modification mediates the biogenesis of circDDIT4, which binds to ELAVL1 and acts as an RBP sponge to downregulate the expression of ELAVL1 target mRNAs, including ANO7, thus exerting a tumor suppressor effect on the progression of prostate cancer [79]. METTL3 stabilizes ARHGDIA mRNA by regulating the expression of mRNA-binding protein ELAVL1, thereby alleviating the decay of ARHGDIA mRNA [83]. Cai et al. found that the reduction in METTL3 inhibited the SHH-GLI1 signaling axis, decreased mRNA levels of the downstream targets, such as c-Myc and cyclin D1, and significantly decreased PCa cell survival, and inhibited the development of tumors [24].

## Non-coding RNA m6A modifications in prostate cancer

**Fig. 2:**
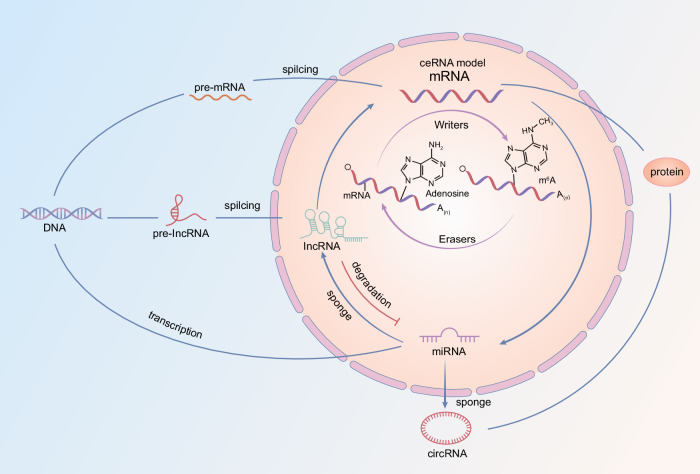
Implications of m6A in different forms of RNAs. The m6A modification targets involved in prostate cancer progression include mRNA and non-coding RNA such as miRNA, circRNA, and lncRNA.The ceRNA network is composed of lncRNA-miRNA-mRNA and circRNA-miRNA-mRNA.

In addition to m6A modifications of mRNA that are functionally critical during prostate cancer initiation, progression, and treatment, other noncoding RNAs’ m6A modifications, such as miRNA, lncRNA, and circRNA are also emerging and reported to be involved in various aspects of prostate cancer (Fig. 2).

### MiRNA m6A modification in cancer

MiRNA is a group of ncRNA that has been proven to be functionally critical during PCa development and progression. Recent accumulating studies have demonstrated the indispensable roles of m6A in miRNAs for their function execution in cancer. MiR-96/182/183 cluster regulated the patient survival and tumor proliferation in melanoma and breast cancer [84, 85]. The upregulation of miR-182 significantly enhanced the migration and invasion of PCa by regulating the expression of FOXO1 or activating the Wnt/β-catenin signaling pathway [86, 87]. Further studies indicated that METTL3 knockdown reduced the expression of miR-182 in PCa in a m6A-dependent manner. Additionally, miR-182 inhibitors can reduce cell proliferation promoted by METTL3 overexpression to a certain extent, and METTL3 is required for DGCR8-regulated pri-miRNA in PCa [88], as the reader protein complex recognizes and processes pri-miRNA, thereby producing mature miRNA that affects cellular processes [89]. Recent studies also reported that MiR-330-3p plays an anti-tumor role in many cancers, including PCa [90–92]. Knocking out HNRNPA2B1 inhibited the interaction between DGCR8 and pri-miR-93, while knocking down METTL3 reduced the level of miR-93-5p and promoted the accumulation of pro-mir-93. In general, HNRNPA2B1 facilitated the processing of pri-miR-93 by recruiting DGCR8 in an m6A-dependent manner [83]. Overexpression of METTL3 can significantly improve the level of miR-148a-3p by promoting m6a modification of pri-miR-148-3p [93]. Additionally, other miRNAs, including miR-320d, miR-495, and miR-139-5p, are also demonstrated to be regulated by m6A and involved in prostate cancer progression. For instance, the overexpression of miR-320d downregulates the mRNA expression of METTL3 and also reduces the expression level of KIF3C, thus delaying the proliferation, invasion, and migration of PCa [94]. KDM5A can promote the proliferation, invasion, and migration of PCa cells and reduce cell apoptosis by down-regulating the expression of miR-495 [95]. METTL1 leads to improved mRNA stability of CDK14 by adding m7G modifications inside its mRNA, finally promoting CRPC progression [96]. Azhati et al. found that in FTO overexpressing PCa cells, inhibition of miR-139-5p and overexpression of ZNF217 could inhibit cell proliferation, mitosis, and EMT [97]. Moreover, FTO inhibited the expression of ZNF217 in a mir-139-5p-dependent manner in PCa cells [97]. In summary, accumulating studies emphasize that m6A modification of miRNAs is closely involved in PCa, which may serve as potential therapeutic targets in the future with the development of RNA targeting technologies.

### LncRNA m6A modification in cancer

More and more studies have shown that lncRNAs can be used as cancer prognostic and diagnostic markers, providing new strategies for cancer prevention and treatment. Abnormal changes of lncRNA have been found in many types of cancer, such as lung cancer, stomach cancer, colon cancer, and PCa [98–101]. Liu et al reported that m6A induced upregulation of lncRNA small nucleolar RNA host gene 7 (SNHG7), which was significantly upregulated in PCa tissues and cells, and accelerated glycolysis through the serine/arginine-rich splice factor 1 (SRSF1)/c-Myc axis and promoted the progression of PCa [102]. In addition, SNHG7 expression was higher in AR inactive PC-3 and DU-145 cells compared to AR-active LNCaP and VCaP cells, and the upregulation of METTL3 increases the stability of SNHG7 in PCa [103]. Besides, METTL3 could upregulate the m6A level of LncRNA-MALAT1, which activates the PI3K/AKT signaling pathway and promotes the growth and invasion of PCa [104]. The enhanced expression of lncRNA (HOXD-AS1) in CRPC cell lines can regulate chemotherapy resistance and proliferation by interacting with the WDR5/MLL1 complex, thereby promoting CRPC transformation [105]. Furthermore, lncRNA SNHG11 expression level is also upregulated in PCa tissues and cells and promotes PCa progression through modification of miR-184 and upregulation of IGF-1R expression in an m6A-dependent manner [106]. LncRNA can also act as a miRNA sponge, inhibit the degradation of target genes mediated by relevant miRNAs, reshape chromatin to regulate the activity of transcription regulators, and bind to RNA-binding proteins to control RNA stability in m6A-dependent manners [107–110].

### CircRNA m6A modification in cancer

As competitive endogenous RNAs, circRNA has been shown to perform multiple functions, especially in cancer, such as displaying a specific spatial and temporal expression pattern [111]. The structure of circRNA lacks free 5 and 3 ends, making it more stable than linear RNA, which is more suitable for serving as a biomarker, compared with other types of RNA [112]. Recent studies have confirmed that some circRNAs play a crucial role in the progression of PCa and have uncovered the potential molecular mechanism of circRNAs with m6A modifications in PCa [103, 113]. Ding et al. reported that circRNA midline-1 (circ-MID1) was overexpressed and functioned to sponge miR-330-3p in PCa cells and promoted the progression of PCa by regulating the miR-330-3p/YTHDC2/IGF1R/AKT axis [114]. Yu et al. reported that overexpressed circ-0003258 could bind to IGF2BP3, increase HDAC4 mRNA stability, and activate the ERK signaling pathway, thereby accelerating PCa metastasis [115]. CircABCC4 interacted with IGF2BP2 protein to upregulate CCAR1 and subsequently repressed the expression of target genes in the Wnt/β-catenin signaling pathway [116]. Additionally, data showed that circRBM33 was m6A-modified and highly expressed in PCa than in normal cells/tissues, and enhanced circRBM33 m6A modification promoted tumor growth and invasion, and decreasing the m6A level rescued the tumor-promoting effect of circRBM33 [117]. Growing evidence shows that circRNA plays an important role in the occurrence and progression of PCa, especially in driving the progression of CRPC [118]. Both lncRNA and circRNA can prevent the degradation of targeted mRNA by sponging miRNA [119, 120]. For example, circ-TRPS1 inhibits the growth, invasion, and migration of CRPC cells by sponging miR-124-3p to enhance EZH2 expression [121]. Further studies are needed to reveal the interaction network and regulation of mRNA and ncRNA, as well as the implications of m6A modification in cancer.

## Strategies to target cancer therapy through m6A

Given the close relation of m6A dysregulation with tumorigenesis and malignancy progression [18, 122–124], targeting m6A regulators or the oncogenic m6A events holds huge potential for PCa treatment. Over the past few decades, there have been several advances in targeted cancer therapy, including many anti-cancer drugs and emerging technologies based on transcription and post-transcription levels by targeting m6A modifications in cancer.

### Small molecules targeting m6A regulators hold therapeutic potential for prostate cancer

Given that the dysregulation of m6A in cancer can promote the development of malignant tumors, more and more small molecules have proven to be effective in suppressing prostate cancer by targeting m6A (Table 1). Specifically, Feng et al. found that β-elemive can also reduce the m6A modification level of PTEN mRNA by down-regulating the expression of METTL3, leading to increased expression of PTEN protein, and ultimately inhibiting the growth of tumor cells and promoting cell apoptosis [125]. Patients with metastatic prostate cancer exhibiting PTEN loss tend to have longer imaging-based progression-free survival and are more frequently associated with advanced AJCC stages [28, 48]. Another small molecule, STM2457, a METTL3 inhibitor, suppresses the proliferation, migration, and invasion of prostate cancer cells by reducing m6A methylation levels. It also exhibits synergistic effects with the PARP inhibitor Olaparib to further inhibit prostate cancer progression [126]. FB23-2 is a promising inhibitor by targeting FTO, which mediates the demethylation of m6A of FB23-2 and induces inhibition of cell proliferation and activation of apoptosis in AML cells [127]. IGF2BP2, an m6A binding protein that enhances the stability and translation of mRNA, is highly expressed in acute myeloid leukemia AML cells and promotes leukemia progression by regulating the expression of MYC. Its expression is upregulated in prostate cancer tissues with distant metastasis, indicating its potential as a therapeutic target [128, 129]. ALKBH5 deficiency in macrophages increases m6A modification on IL-11 mRNA, which results in decreased IL-11 stability [130]. Susanne M. et al. found that long-term consumption of quercetin and green tea extract in prostate cancer patients enhanced the bioavailability of polyphenols and reduced methylation activity [131]. A potent METTL3 selective inhibitor (UZH2, METTL3 IC_50_ = 5 nM) has certain inhibitory activity in the MOLM-13 cells (EC_50_ = 0.7 μM) and prostate cancer cells (EC_50_ = 2.5 μM) [132]. FTO signal transduction promotes UUO/ TGF-β1-induced rise of RUNX1 in vivo and in vitro by demethylating RUNX1 mRNA and improving its stability [133]. TGF-β expression was downregulated and bone morphogenetic protein (BMP-2) was enhanced in castrated SCID mice xenografted with C4-2B cells injected into the tibia and treated with curcumin, but downstream signaling was not affected [134]. Treatment of LNCaP cells with curcumin can induce a change in their methylation status and upregulate the expression of HDAC4 in the cells [135]. After curcumin treatment of 22Rv1 cells, the expression of MIR-141 and MIR-183 was enhanced, but the expression of the androgen receptor (AR) was decreased [136]. Previous studies confirmed that baicalin could also be used to treat various disorders by regulating methylation in key genes [137–139]. Baicalin improved mouse embryo development in vitro by improving DNA methylation, inhibiting cellular apoptosis, and modulating HSP70 expression [138]. Moreover, baicalin promoted suv39h1 splicing by enhancing RNA m6A methylation, resulting in anti-nasopharyngeal carcinoma (NPC) behavior [137]. The expression of FOXM1 in LNCaP and PC3 cells of the baicalin-treated group was significantly lower than that of the control group [140]. Baicalin treatment decreased the expression level of NF-κB p-P65 protein in xenograft tumors [141].

**Table 1:** Small molecules and their targets in prostate cancer.

Small molecules	Target	Experiment model	Reference
β-elemive	METTL3	Patients with Nonmetastatic Prostate Cancer	[169]
STM2457	METTL3	PC3,22RV1,DU145	[126]
IGF2BP2	MYC	Patients with prostate cancer	[128, 132]
UZH2	METTL3	PC-3	[133]
curcumin	TGF-β	SCID mice implanted with C4-2B	[134]
	HDAC4	LNCaP	[135]
	AR	22Rv1	[136]
	MIR-141		
	MIR-183		
baicalin	FOXM1	LNCaP PC3	[140]
	NF-κB p65	In situ PCa stem cells (PCSCs) injected BALB/c nude mice	[141]

### Natural products derived from traditional medicine for targeting m6A

Accumulating evidence indicates the emerging role of natural products in anti-tumors, especially by targeting m6A (Table 2), such as phenols [142–144], flavonoids [137, 145–147], alkaloids [148], and anthraquinones [149–151]. For example, Curcumin, a phenolic compound, inhibited adipogenesis by reducing the expression of ALKHB5 and led to higher m6A modification of TNF receptor-associated factor 4 (TRAF4) mRNA [142]. Epigallocatechin gallate (EGCG) is a polyphenol present in green tea (*Camellia sinensis*), which has revealed anti-cancer effects toward a variety of cancers in vitro and indicated protective potential against neurodegenerative diseases such as Alzheimer’s and Parkinson’s [152]. EGCG decreases FTO protein expression, which leads to increased m6A-modified CCNA2 and CDK2 mRNA and has a significant effect on inhibiting obesity and adipogenesis [147]. Ren et al found that the camptothecin (CPT) analogs caused the fluorescence quenching of FTO through a static quenching procedure [153]. Given the oncogenic role of FTO in cancers, these agents may also exert anti-tumor efficacy, though further experiments are required. Recent studies confirmed that baicalin hydrate(BH), a natural product derived from traditional Chinese medicines (TCM), could also be used to treat various disorders by regulating methylation in key genes’ mRNA [137–139]. Baicalin promoted suv39h1 splicing by enhancing RNA m6A methylation, resulting in anti-nasopharyngeal carcinoma (NPC) behavior [137]. Elemene, a natural hemiterpenoid compound, is an extract of turmeric and is a mixture of β-, γ-, δ-elemene, with β-elemene as the main component. β-emolene could directly inhibit the expression of METTL3 and lead to the downregulation of autophagy-related proteins LC3B, ATG5, and ATG7, thereby inhibiting autophagy, tumor cell proliferation, and promoting cell apoptosis [154]. Although most of these compounds lack direct evidence for anti-tumor effects in prostate cancer specifically (despite efficacy in other cancers), they hold great promise for prostate cancer therapy. This potential stems from their ability to target m6A regulators and m6A-modified oncogenes known to drive prostate cancer, highlighting the need for focused future research.

**Table 2:** Natural products and their targets in various disorders.

Reagent	Target	Disease	Effect in the pathway	Reference
Curcumin	ALKHB5	Obesity	Reduce ALKHB5 expression and increase TRAF4 mRNA; anti-obesity	[141]
EGCG	FTO	Obesity	Decrease FTO protein expression, increase CCNA2 and CDK2 mRNA m6A modification; inhibit obesity and adipogenesis	[146]
CPT			Caused the fluorescence quenching of FTO through a static quenching procedure	[152]
BH	Sat2, α-Sat2, Major-Sat	NPC	promote suv39h1 splicing by enhancing RNA m6A; anti-NPC	[134]
Elemene	METTL3	Autophagy apoptosis	Inhibit METTL3 expression, downregulate autophagy-related protein LC3B, ATG5 and ATG7; inhibit autophagy and cell proliferation, promote cell apoptosis	[153]

### CRISPR-Cas systems targeting m6A regulators and oncogenic m6A events

**Fig. 3:**
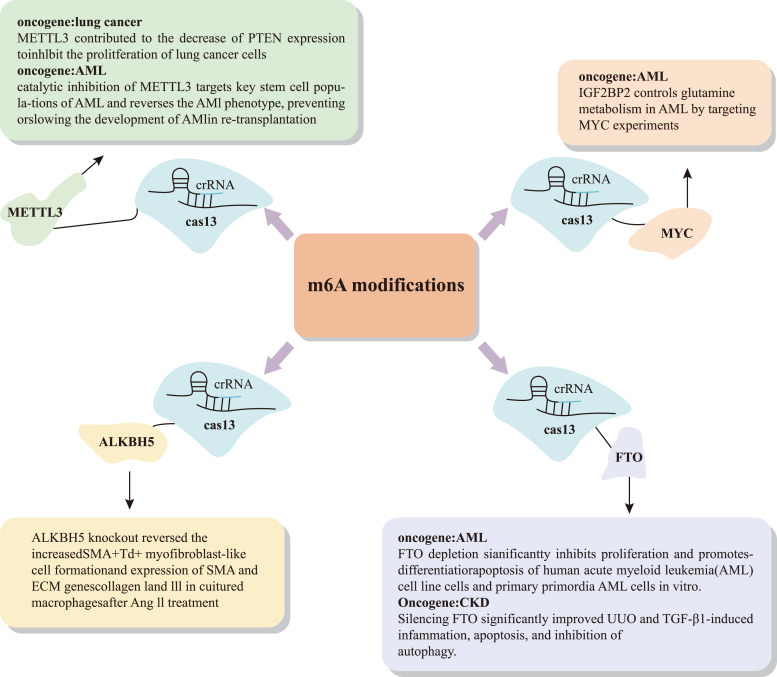
The emerging roles of CRISPR-Cas13 in regulating m6A in cancer. In various cancer models—including lung cancer, acute myeloid leukemia (AML), and chronic kidney disease (CKD)—the expression of downstream molecules is influenced by targeting m6A regulators or m6A modifications. It is therefore reasonable to hypothesize that related epigenetic changes, both in vivo and in vitro, can be detected using CRISPR-Cas13–based systems.

In addition to the small agents modulating the m6A for the treatment of cancer, emerging technologies such as CRISPR-Cas9 and CRISPR-Cas13 [155–157] are being used in targeting m6A for tumor therapies. For example, unlike traditional chemotherapy drugs that damage normal cells, gRNA-guided RNA-targeting CRISPR-Cas13 is of great help in cancer diagnosis, treatment, and research due to its high specificity and efficiency in targeting RNA m6A (Fig. 3) [158]. CRISPR-Cas13-based biosensors have been used to efficiently and specifically detect cancer markers such as circulating tumor DNA, tumor-derived RNA, and as well as m6A-modified RNAs from liquid biopsy samples [159, 160]. CRISPR-Cas13 has also been used to efficiently and specifically target a variety of cancer-associated RNA and RNA modifications, such as m6A in vitro and in vivo [161]. Unlike the DNA-targeting activity of CRISPR-Cas9 and CRISPR-Cas12, CRISPR-Cas13 systems are the first and only RNA-targeting CRISPR-Cas systems that exclusively cleave ssRNA to date [162]. A-to-I editing is a significant post-transcriptional modification mediated by the ADAR family of enzymes. ADAR1 has recently been identified as a downstream target of METTL3 and has been shown to promote the progression of glioblastoma [163]. Moreover, elevated ADAR1 expression is associated with the advancement of castration-resistant prostate cancer (CRPC), highlighting its potential as a therapeutic target[171]. To enable programmable RNA editing, Feng Zhang’s team developed the REPAIR system, which fuses catalytically inactive Cas13 with the deaminase domain of ADAR [164]. This RNA-targeting platform not only facilitates precise A-to-I editing but also holds promise for identifying novel therapeutic targets in prostate cancer. In addition, several RNA manipulation tools based on CRISPR-Cas13 have been developed [160, 161, 165]. Lekha Nair et al. have employed dCas13b-FTO and dCas13b-ALKBH5 to demethylate the m6A modification on SμGLT and found that m6A can catalyze the function of CSR [162]. Zheng et al. speculated that the CRISPR-Cas13-mediated m6A editing platform may also be an effective tool to improve the antiviral ability of crops [166].

Sigitas et al. developed the meCLICK-Seq technique to capture extensive methylation of low-abundance transcriptome regions at high resolution and low RNA initiation in introns and intergenic regions, identifying RNA-modified substrates. M6A is widely present in intergenic regions of lncRNA and introns, mainly deposited by METTL16. This discovery provides new ideas for the development of new technologies that target the degradation ability of nucleic acids [166]. Jie et al. found that PAMECR can provide an efficient and precise spatiotemporal control of m6A editing and manipulate cell differentiation. It also provided a basis for the development of improved gene and non-coding RNA manipulation tools and control of cells at the surface transcriptome level [166]. Zhao et al. engineered an optogenetic system, PAMECR, based on CRISPR-Cas13 and light-inducible heterodimers (CIBN-dCas13/CRY2PHR-m6A effector), enabling spatiotemporally controlled m6A editing via blue light and allowing multiplexed editing and near-infrared operation via upconversion nanoparticles for therapeutic epitranscriptome engineering [159]. These studies collectively demonstrated great potential for m6A-targeted therapy with CRISPR-Cas-based platforms.

## Discussion and perspective

Epigenetic modifications include DNA methylation, histone modifications, and RNA modifications, which have unique roles and mechanisms in the development of PCa. m6A is the most prevalent epigenetic modification in various RNA types and plays an important role in promoting the development of PCa. m6A-based epigenetic events in PCa have emerged as a key player in PCa development, progression, and treatment resistance [167]. Two kinds of molecules may serve as anti-tumor targets in the m6A process in PCa, the m6A regulators including “readers”, “erasers”, and “readers”, which regulate the stability, metabolism, and maturation of cancer-related mRNA and ncRNA, and the oncogenic m6A modification events including mRNA and ncRNAs such as miRNA, circRNA and lncRNA. Future studies are needed to further reveal the roles and mechanisms of m6A regulators and their downstream m6A modification-regulated targets in PCa for helping to screen the specific targets in different circumstances for cancer treatment [168]. It is worth noting that, natural products derived from traditional medicine, as well as the emerging RNA editing technologies, especially CRISPR-Cas13/dCas13, hold great promise for PCa treatment by targeting m6A regulators and m6A events.
